# Molecular typing of the recently expanding subtype B HIV-1 epidemic in Romania: Evidence for local spread among MSMs in Bucharest area^☆^^☆☆^

**DOI:** 10.1016/j.meegid.2012.03.003

**Published:** 2012-07

**Authors:** Simona Paraschiv, Dan Otelea, Ionelia Batan, Cristian Baicus, Gkikas Magiorkinis, Dimitrios Paraskevis

**Affiliations:** a Molecular Diagnostics Laboratory, ‘Prof. Dr. Matei Bals’ National Institute for Infectious Diseases, Str. Calistrat Grozovici, Nr. 1, Sector 2, 021105 Bucharest, Romania; b Department of Internal Medicine, Colentina Hospital, Bucharest, Romania; c National Retrovirus Reference Center, Department of Hygiene and Epidemiology, Faculty of Medicine National and Kapodistrian, University of Athens, Greece; d Department of Zoology, University of Oxford, Oxford, United Kingdom

**Keywords:** HIV-1 Romanian epidemic, Subtype B, Molecular epidemiology, Phylogenetic analysis

## Abstract

HIV-1 subtype B is predominant in Europe except in some countries from Eastern Europe which are characterized by a high prevalence of non-B subtypes and circulating recombinant forms (CRFs). Romania is a particular case: the HIV-1 epidemic started with subtype F1 which is still the most prevalent. Previous studies have shown an increasing prevalence of subtype B which is the second most frequent one among the newly diagnosed individuals, followed by subtype C and several CRFs as well as unique recombinant forms (URFs). Our objective was to analyze in detail the characteristics (way of dispersal, association with transmission risk groups) of the subtype B infections in Romania by means of phylogenetic analysis. Among all the individuals sampled during 2003–2010, 71 out of 1127 patients (6.3%) have been identified to be infected with subtype B strains. The most frequent route of infection identified in HIV-1 subtype B patients in Romania was MSM transmission (39.6%), followed by the heterosexual route (35.2%). Many of the patients acquired the infection abroad, mainly in Western European countries. Phylogenetic analysis indicated the existence of a local transmission network (monophyletic clade) including 14 patients, mainly MSM living in the Bucharest area. We estimate the origin of the local transmission network that dates at the beginning of the 90s; the introduction of the F1 and C subtypes occurred earlier. The rest of the sequences were intermixed with reference strains sampled across Europe suggesting that single infection were not followed by subsequent dispersal within the local population. Although HIV-1 subtype B epidemic in Romania is recent, there is evidence for local spread among the MSMs, in addition to multiple introductions.

## Highlights

► The most frequent route of infection in subtype B patients was MSM transmission. ► Many of the patients acquired the infection abroad, in Western European countries. ► A local transmission network has been identified. ► This network includes mainly MSM living in the Bucharest area. ► Subtype B local transmission network is recent, dating at the beginning of the 90s.

## 1 Introduction

Human immunodeficiency virus type 1 (HIV-1) infection continues to spread throughout the world despite a downward trend of HIV new infections; an estimated 33.3 million people are currently living with this virus (UN Joint Programme on HIV/AIDS, Global Report: 2010).

The genetic diversity of HIV-1 caused by its high mutation and recombination rate (Wain-Hobson, 1989; Onafuwa-Nuga and Telesnitsky, 2009) is reflected by the existence of several types and subtypes: four distinct genetic groups have been described so far: M (major), O (outlier), N (non-M, non-O) and the newly discovered P group (Plantier et al., 2009). The largest group of HIV-1 sequences is M, which includes nine ‘pure’ subtypes (A, B, C, D, F, G, H, J and K), sub-subtypes (A1–A4 and F1–F2) (Robertson et al., 2000; Paraskevis and Hatzakis, 1999), 49 inter-subtype circulating recombinant forms (CRFs) and several unique recombinant forms (URFs) (http://www.hiv.lanl.gov/content/sequence/HIV/CRFs/CRFs.html). Most subtypes and CRFs are present in Africa, the origin place of HIV-1 epidemic (Gao et al., 1999). Almost half of the total infections are caused by the subtype C strains, whereas subtype B is the most frequent in America, Western and Central Europe, in some areas of Asia and Australia (Buonaguro et al., 2007; Hemelaar et al., 2006, 2011). The HIV-1 molecular epidemiology is more complex in Europe than in America, varies geographically and is influenced by transmission risk and social factors (Marcus et al., 2006; Strathdee et al., 2010; Platt et al., 2011). In Western and Central Europe, subtype B is the most common and is mainly observed in men who have sex with men (MSM) and intravenous drug users (IVDUs). Lately, the number of non-B subtypes has constantly increased in Western Europe, being often associated with heterosexual transmission and/or immigration. This trend has been observed in Austria, Belgium, Greece (subtype A), Sweden, Denmark, United Kingdom (subtype C), Portugal (subtype G) (Snoeck et al., 2004; Vercauteren et al., 2009; Esteves et al., 2003).

In several regions of Central and Eastern European countries non-B subtypes and circulating recombinant forms (CRFs) have occasionally been found having high prevalence. Subtype A is common in countries such as Belarus, Ukraine, Russia, Bulgaria and the former Yugoslavia (Saad et al., 2006; Lazouskaya et al., 2005; Lukashov et al., 1999; Salemi et al., 2008a; Stanojevic et al., 2002). A significant presence of CRF01_AE has been recently reported in Bulgaria (Alexiev Ivo, personal communication) while CRF03_AB is prevalent in the Kaliningrad enclave (Bobkov et al., 2004). Romania is a particular case: the HIV-1 epidemic started with the subtype F1, which is still the most prevalent one, however the epidemic has changed over time. In early 90s, the HIV cases were mainly in the nosocomially infected pediatric population (Hersh et al., 1993; Op de Coul et al., 2000), while in more recent years the newly diagnosed patients have been mostly heterosexually infected young adults (24–35 years). Previously we reported an increasing trend of subtype B strains from 0% of the total genotyped sequences in 2003 to 7.8% in 2007 (Paraschiv et al., 2008). This subtype is the second most frequent among the newly diagnosed individuals, followed by subtype C and several CRFs as well as URFs (Florea et al., 2010). The objectives of this study were to analyze in detail the epidemiology of subtype B infections in Romania (origin, way of dispersal, potential association with transmission risk groups) and the timeline of this sub-epidemic.

## 2 Methods

### 2.1 Study population

We analyzed 71 samples from individuals infected with HIV-1 subtype B referred to the reference laboratory from the National Institute for Infectious Diseases ‘Matei Bals’. These patients comprise approximately 6.3% of the total number of subjects (*N* = 1127) investigated between 2003 and 2010 as part of drug resistance testing in Romania. The samples were collected in all nine HIV regional centres that cover evenly the Romanian territory and population. The percentage of the patients infected with this particular subtype was slightly higher, 8.2% (19 out of 232), when only newly diagnosed (2007–2009) individuals were considered.

The blood samples have been obtained from naïve (*n* = 22, 31%) and treated patients (*n* = 49, 69%). Epidemiological and demographic data for the study population are listed in Table 1. To compare the patient groups carrying different HIV-1 subtypes, categorical variables were analyzed with Fisher’s exact test (InStat 3.0, http://www.graphpad.com). The study has been conducted according to the current local ethical regulations.

### 2.2 RT-PCR and HIV-1 subtyping

The plasma samples were obtained from EDTA-whole blood (centrifugation at 3000 rpm for 15 min) and stored at −80 °C prior to genotypic testing. RNA was extracted from 500 μl of plasma using the sample extraction module of the commercial kit Viroseq™ HIV-1 Genotyping System (Celera Diagnostics, Alameda, CA), according to the manufacturer’s recommendations. RT-PCR was performed on a GeneAmp System 9700 (Applied Biosystems) thermal cycler, following the manufacturer’s recommendations. The amplified products, 1.8 kb in length (the full protease sequence and two-thirds of the reverse transcriptase), were purified and sequenced bidirectionally on an ABI Prism 3100-Avant Genetic Analyzer (Applied Biosystems) using Big Dye Terminator chemistry and six different primers. The raw analysis of the sequences was made using Sequencing Analysis Software Version 3.7 (Applied Biosystems); they were then assembled with ViroSeq 2.5/2.7/2.8 HIV-1 Genotyping System Software (Celera Diagnostics, Alameda, CA).

HIV-1 subtyping was performed using the publicly available algorithm REGA HIV-1&2 Automated subtyping tool version 2.0 (http://www.jose.med.kuleuven.be/genotypetool/html/indexhiv.html).

### 2.3 Reference strains

A large set of sequences sampled across Europe and available from public databases (7,157 sequences) were selected using the geographic distribution of HIV-1 sequences tool available at the Los Alamos HIV Sequence Database (http://www.hiv.lanl.gov/components/sequence/HIV/geo/geo.comp). The countries that were reported as possible source for the infection in this study (Spain, Italy, Germany, Netherlands, United Kingdom) were adequately represented in the reference group of sequences. The final dataset analyzed consisted of the 71 studied sequences together with 155 subtype B reference sequences from most of the European countries. The country distribution of these sequences and the accession numbers are listed in the Supplementary Table. The HIV-1 subtype D sequences that were used as outgroup have the following accession numbers: AY253311, U88824, U88822, AY371157.

### 2.4 Molecular typing of the epidemic

The identification of local transmission networks was performed as following: the reference sequences were aligned along with the Romanian subtype B sequences using CLUSTAL W as implemented in BioEdit software version 7.0 (http://www.mbio.ncsu.edu/BioEdit/bioedit.html). To avoid convergent evolution bias under antiretroviral selective pressure, the phylogenetic analysis was performed after excluding all major resistance sites in protease and reverse transcriptase as described previously (Paraskevis et al., 2009).

Phylogenetic analysis was performed using maximum likelihood as implemented in PAUP^∗^ (Swofford, 1998), using the GTR (general time reversible) as nucleotide substitution model and gamma (Γ) distribution of rate variability among sites, calculated empirically from the data with six categories of rates. Neighbor joining trees were built on bootstrapped alignments (1000 replicates) to assess the robustness of the obtained topologies. Transmission networks were assigned as those clades consisting of at least four sequences of Romanian origin receiving >75% bootstrap support. Phylogenetic trees were visualized using the FigTree version 1.3.1 program (http://www.tree.bio.ed.ac.uk/software/figtree/). All sequences were screened for hypermutation using the Hypermut 2.0 (http://www.hiv.lanl.gov/content/sequence/HYPERMUT/hypermut.html) and for inter-subtype recombination using RIP (http://www.hiv.lanl.gov/content/sequence/RIP/RIP.html) with a window size of 100.

### 2.5 Molecular clock analysis

The sequences used for molecular clock analysis were of strains isolated from several naïve patients infected with the subtype B. We assembled a dataset with Romanian sequences and reference sequences with known sampling dates, available in the Los Alamos database. The reference dataset included 18 sequences sampled from North, South America, Europe and Australia. To maximize the sampling window and increase the information needed to estimate the molecular clock rate we selected some of the earliest sampled sequences available in the database. Specifically, the temporal range of sampling in the reference datasets was between 1983 and 2007. Phylodynamic analysis was performed using a Bayesian approach as implemented in BEAST version 1.5.1 (Drummond and Rambaut, 2007) using the GTR model of nucleotide substitution, assuming Γ-distributed rates among sites and invariable sites. We used as coalescent tree priors the uncorrelated lognormal relaxed clock model (Drummond et al., 2006) with TipDates and Bayesian skyline with 10 number of groups (Drummond and Rambaut, 2007). Two separate Markov chain Monte Carlo (MCMC) runs were made for 10^7^ generations with a burn in of 10^6^. MCMC was sampled every 1000 generations. No additional priors were used in the analysis. We assessed convergence of the Markov chain (ESS > 100) by means the program Tracer v1.4 (http://tree.bio.ed.ac.uk/software/tracer/). The consensus for each run was inferred by the TreeAnnotator program (Drummond and Rambaut, 2007).

### 2.6 Robustness and justification of model selection

There were two reasons why we used the uncorrelated lognormal relaxed clock model: the number of coefficient of variation estimates in the analysis was high (median estimate: 0.315; 95% HPD: 0.12–0.54) and we preferred making no assumptions on the molecular clock behavior of the virus. As nucleotide substitution model we used the GTR + G + I, which has been shown previously to be the best to fit for protease and partial reverse transcriptase (Hué et al., 2004, 2009; Lewis et al., 2008) and limits to the minimum the assumptions on the nucleotide substitution process. Finally, we used the Bayesian skyline non-parametric demographic model and no assumptions were made on the underlying demography of the epidemic. To sum up we used a very robust phylodynamic framework with the least possible number of assumptions, choosing to be more conservative and loosing power in our estimations than making rough assumptions.

## 3 Results

The majority of the studied patients infected with subtype B strains were men (78.9%, 56/71) and the main route of transmission for this group was through homosexual contact (50%, 28/56). The overall proportion of MSM in the study population was 39.4% (28/71); the statistical analysis showed that it was significantly higher (*p* < 0.0001) in subtype B infected patients than in those infected with other subtypes reported in Romania (F1 and C). The prevalence of homo/bisexual practice was 4.75 (95% confidence interval: 3.46–6.5) times higher among men with subtype B than among men with other subtypes reported in Romania (*p* < 0.0001). Most of the MSMs were young persons (25–38 years old). However, the heterosexual contact was reported to be responsible for the infection in 35.2% of the studied patients, 14 of them being males and 11 females (Table 1). IVDUs represented a very small proportion of all patients (this risk factor has been reported in five cases). The IVDUs are currently a small albeit rapidly increasing risk group in Romania (http://www.cnlas.ro/images/doc/date_30iunie2011.pdf).

A relatively large number of the patients (33/71, 46.5%) reported they had possibly been infected abroad, in countries from Western Europe. This is in contrast with data on patients infected with subtype C, where only 18.9% (7/37) reported other counties as presumed place of infection. Spain has been indicated as the most frequent foreign country for suspected HIV infection (12 reports), followed by Italy (10 patients), Germany (five cases), Netherlands (four cases), United Kingdom (two patients). Mother to child transmission has been encountered in two cases that were diagnosed in 2002 and 2007 (Table 1).

Twenty two patients were ARV treatment naïve at the time when the blood sample was drawn. A quarter of the studied patients (25.4%, 18/71) were diagnosed in recent years (2007–2010), with recent infections. Among the studied patients, we also observed three declared couples (two of them MSMs, one heterosexual).

**Fig. 1:**
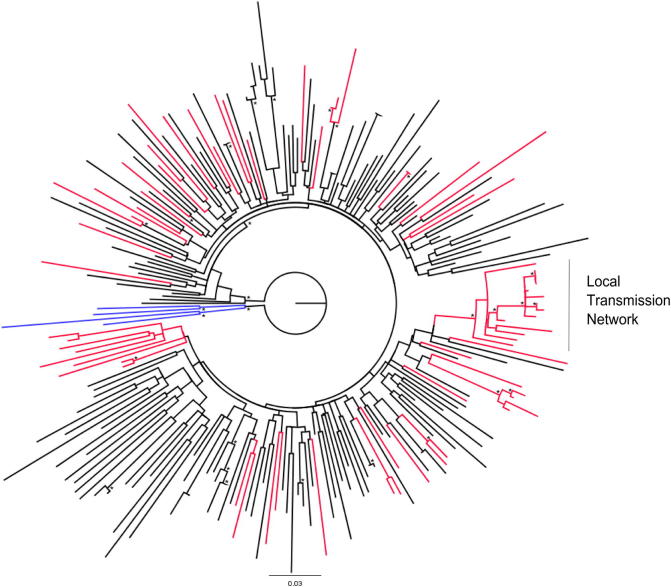
Maximum likelihood phylogenetic analysis of HIV-1 subtype B *pol* sequences. Romanian sequences are marked in red, sequences from other European countries are shown in black. The tree was rooted using four HIV-1 subtype D sequences as outgroup, marked in blue. The bootstrap support values greater than 0.7 are indicated by an asterisk at the nodes.

The phylogenetic tree of Romanian subtype B sequences is presented in Fig. 1. All the local sequences were marked in red. This analysis showed two patterns of subtype B dispersal in Romania. First, through a local transmission network (a group of 14 patients) as indicated by the monophyletic clade, distinctly marked in the tree. The corresponding strains were isolated from 14 patients (13 men), mainly MSM (11/14, 78.6%), most of them living in the Bucharest area and having limited contacts outside the country. The characteristics of these patients are summarized in Table 2. The monophyletic cluster identification suggests a single or limited subtype B strains introduction followed by their spread within the local population at a particular risk (e.g. homosexual contact). We also observed a second cluster, less extended, composed of only four sequences. The corresponding patients are all men and three of them reported the MSM contact as responsible for the HIV infection.

The second pattern is consistent with multiple introductions as reflected by high levels of dispersal across the tree. The phylogenetic analysis revealed high levels of mixing of Romanian sequences with reference strains sampled across Europe. Single clades suggest imported infections not followed by subsequent dispersal within the local population. Some limited clustering of the sequences could be observed, being restricted to sexual couples, vertical transmission cases and two IVDUs.

**Fig. 2:**
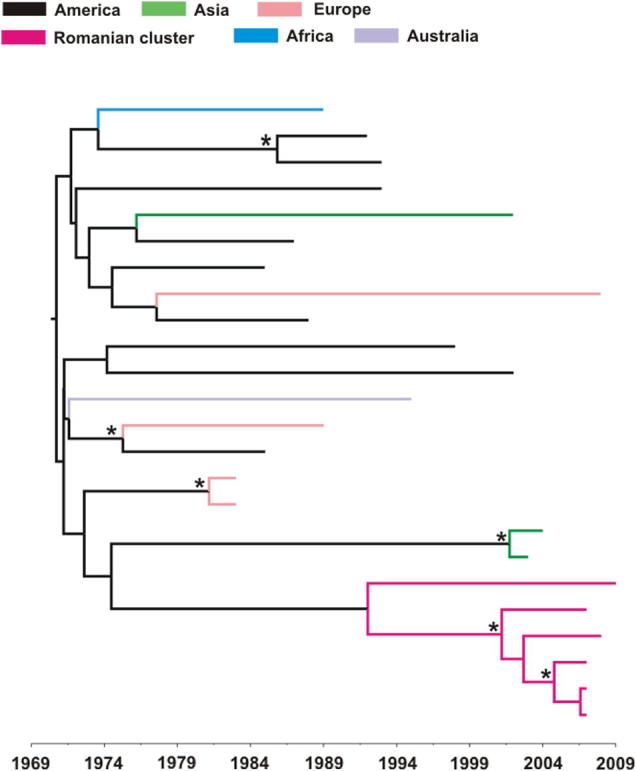
Molecular clock analysis of HIV-1 subtype B *pol* sequences. The Romanian sequences analyzed are part of the identified local transmission network. The tree was generated as described under Section 2. The internal nodes labels represent the posterior probability support (only values higher than 0.9 are indicated by an asterisk). The Romanian sequences branches are marked in magenta. The scale is in years.

In order to estimate the time to most recent common ancestor (tMRCA) of the HIV-1 subtype B monophyletic cluster, we performed a molecular clock analysis, using the sequences from six untreated patients, all MSMs (Fig. 1). The tMRCA inferred for subtype B MSM transmission network was in 1991 (95% Higher Posterior Density HPD: 1983–1999), whereas the tMRCA of the split of lineages for most infections within the cluster was much more recent (median estimate: 2001; 95% HPD: 1997–2004). The result of the molecular clock analysis is shown in Fig. 2.

## 4 Discussion

Our findings reflect some particular aspects of the HIV-1 subtype B epidemic in Romania. The most encountered risk factor for this particular subtype was the MSM transmission, followed by heterosexual transmission. Epidemiological data report MSM as 10.4% of the patients diagnosed in 2010 (http://www.cnlas.ro/images/doc/romania31dec_eng.pdf). Although not very high, this figure is part of an increasing trend (7.9% and only 1.8% of the new HIV cases reported in 2009 and 2006, respectively) possibly reflecting a decreased reluctance to disclose this sexual orientation during recent years; overall, this sexual behavior risk factor is linked to only 1.78% of the accumulated HIV/AIDS cases in Romania (http://www.cnlas.ro/images/doc/romania31dec_eng.pdf).

We found that the MSMs are at five times higher risk of carrying a subtype B strain than either subtype F1 or C. This particular subtype is increasingly encountered in Romania: a quarter of the subtype B infected patients were recently diagnosed persons (2007–2010). Furthermore, many of the subtype B patients reported that they acquired the infection outside the country, especially in Western European countries, in contrast with the Romanian patients carrying subtype F1 and C strains, which were infected mostly locally (Paraschiv et al., 2011).

The phylogenetic analysis of HIV-1 subtype B revealed two distinct patterns of spread. We identified a single large MSM Bucharest-specific cluster, suggesting that the infection among members of this population was introduced by one founder event. The rest of viral isolates formed single branches or very small clades intermixed with reference sequences from other European countries, mainly from the west of the continent. This pattern suggests that single introductions were sometimes followed by limited dispersal, an observation that is in concordance with the patient-reported possible origin of infection.

Several studies estimating the date of HIV-1 epidemics in different geographical parts of the world have been done so far (Robbins et al., 2003; Gilbert et al., 2007; Hué et al., 2005; Salemi et al., 2008b). An estimation for the origin date of US subtype B epidemic around 1968 was made by Korber et al. and subsequently confirmed by Robbins et al. (Korber et al., 1998; Robbins et al., 2003). The first subtype B migration event out of Africa is thought to have occurred around 1966 in Haiti (Gilbert et al., 2007). The virus then moved around 1969 to US (1966–1972) (Lukashov and Goudsmit, 2002). From US, the HIV-1 subtype B was introduced in Europe mainly through MSM transmission and/or IVDUs (Glauser and Francioli, 1984; Lukashov et al., 1996).

The molecular clock analysis of Romanian subtype B sequences from the identified local transmission network showed that its origin is rather recent. The tMRCA of the Romanian local transmission chain was inferred in 1991 (median estimate; 95% HPD: 1983–1999) and the date of coalescent events for five out of six cases within the cluster was estimated to occur in 2001 (median estimate; 95% HPD: 1997–2004). These findings suggest that the epidemic spread of subtype B within the local network probably started 10 years ago and are compatible with our previous analysis showing an extended spread of subtype B in Romania in recent years (Paraschiv et al., 2008). Given the limited number of available sequences, the estimated tMRCA should be considered as the lower bound for tMRCA of the Romanian cluster.

A recent study assessed the date of origin for subtype F1 epidemics in Romania using phylogeographic analysis (Mehta et al., 2011). They estimated that the tMRCA for Romanian F1 subtype was in 1978 (1972–1983), while for the Angola F1 strains was 1975 (1968–1980). They also suggested that subtype F1 strains were introduced from Angola to Romania through multiple import events in the late 80s and extensively spread into the pediatric population.

Notably, molecular clock analysis of the Romanian subtype C sequences indicated that they were introduced in Romania one decade before the subtype B strains, at approximately the same time with subtype F1 sequences: we estimated the tMRCA for the subtype C sequences spreading mainly among heterosexuals to be around 1981 (data unpublished). These findings agree with our previous study that showed subtype C strains were present in Romania in the late 80s in some nosocomial cases (Paraschiv et al., 2011).

Therefore, subtype B at risk of spreading among MSMs represents the most recent HIV-1 epidemic in Romania and its dispersal occurs in urban areas. These results provide valuable information for the effective control of the epidemic since prevention should focus on the vulnerable population in Bucharest but potentially in other big cities, where we may face similar clusters of subtype B infection. These data are consistent with those reported in other countries: the subtype B epidemic is correlated with MSM as risk factor and clustering is often observed in this population (Chalmet et al., 2010; Kouyos et al., 2010; Bezemer et al., 2010). The particularity of the Romanian case is that, in contrast to the Western hemisphere, the proportion of the B strains in the HIV-1 pool seems to follow an increasing trend. An extremely high prevalence of the F1 subtypes and the parenteral route of transmission were characteristic in the early years of the epidemics. More recent international mobility led to the increased prevalence of B strains.

In conclusion, our findings suggest that subtype B epidemic in Romania is associated with MSM transmission and it was recently introduced to this country, having a complex pattern of dissemination.

## Figures and Tables

**Table 1:** Epidemiological data of the study population.

Characteristic	No. of patients	Percentage
	*n* = 71	%
Age (in years)		
⩽38	40	56.3
>38	31	43.7
Gender		
Male	56	78.9
Female	15	21.1
Route of infection		
MSM	28	39.4
Heterosexual	25	35.2
IVDU	3	4.2
MSM and IVDU	2	2.8
Sexual	2	2.8
Vertical	2	2.8
Blood	2	2.8
Not available	7	9.9
Presumed place of infection		
Romania	25	35.2
Abroad	33	46.5
Unknown/not available	13	18.3
ARV treatment status		
Naïve	22	31
Treated	49	69
HIV diagnosis		
Newly diagnosed (2007–2010)	18	25.4
Early diagnosed (<2006)	53	74.6
Residence		
Bucharest area	28	39.4
Other geographical regions	43	60.6

**Table 2:** Monophyletic cluster (transmission network) of Romanian HIV-1 subtype B: characteristics of the patients, comparison between these patients and the other subtype B infected patients.

Characteristic	Cluster	Other than cluster	*p*-Value	ODDS ratio	95% CI
No. of patients	*N* = 14	*N* = 57			
Age (in years)					
⩽38	9	32	0.763	1.4	0.4–4.7
>38	5	25			
Gender					
Male	13	43	0.273	4.2	0.5–35.3
Female	1	14			
Residence					
Bucharest area	10	18	**0.0125**	5.4	1.5–19.6
Other geographical regions	4	39			
Presumed place of infection					
Romania	7	18	0.18	2.8	0.7–11.0
Abroad	4	29			
Unknown	3	10			
HIV diagnosis					
Newly diagnosed (2007–2010)	4	14	0.742	1.2	0.3–4.5
Early diagnosed (<2006)	10	43			
Route of transmission					
MSM	10	14	**0.0043**	6.6	1.8–24.5
MSM and IVDU	1	1			
Heterosexual	2	23			
Unknown	1	6			
