# Supplementary material for: Molecular typing of the recently expanding subtype B HIV-1 epidemic in Romania: Evidence for local spread among MSMs in Bucharest area
Source: Infect Genet Evol. 2012 Jul;12(5):1052–7. doi: 10.1016/j.meegid.2012.03.003 (PMC3778987; doi:10.1016/j.meegid.2012.03.003)
Supplement: Supplementary data 1 [file mmc1.doc]

**Supplementary table.** Subtype B reference sequences used for the phylogenetic analysis: country distribution

**Country of isolation Accession numbers**

Albania **AY611666**, **AY611672**, **AY611684**, **AY611688**

Austria **AF347214**, **AF347518**, **DQ878531**, **DQ878532**, **FJ936557**

Belgium **AF339004**, **AF338981**, **AJ889843**, **DQ177230**, **DQ177232**, **DQ877759**, **FJ653084**, **EU248460**

Bulgaria **EF517439**, **EF517457**, **EF517462**, **EF517464**, **EF517488**

Cyprus **EU673375**, **EU673382**, **EU673408**, **FJ388934**, **FJ388955**, **FJ388960**

Czech Republic **AY694218**, **AY694233**, **AY694364**, **DQ974864**, **DQ974952**, **EU672570**

Denmark **AJ419453**, **AJ582147**, **AM490025**, **AM490879**, **DQ108366**, **DQ877795**, **EF514706**

France **K03455**, **AF003887**, **AF487122**, **AF487137**, **AJ405967**, **AJ577859**, **AJ577899**, **DQ877930**, **DQ877953**, **DQ878075**, **FJ030643**, **FJ649604**

Germany **AF347140**, **AF347190**, **AF347288**, **AY878668**, **AY878677**, **DQ878276**, **FJ030769**, **DQ878304**, **GQ400800**

Greece **DQ878544**, **DQ878548**, **DQ878559**, **DQ878569**, **DQ878595**, **EF563173**

Greenland **AM285220**, **AM285242**, **AM285267**, **AM937019**, **AM937024**

Ireland **DQ877830**, **DQ877832**

Italy **AF251947**, **AF252026**, **AF376547**, **AF493371**, **AF517266**, **AF517471**, **AY352444**, **AY672455**, **AY855419**, **AY855724**, **AY994341**, **AY995503**, **DQ345170**, **DQ345265**, **DQ369253**, **DQ672623**, **DQ878603**, **EF526205**, **EU019810**, **EU496146**,

**FJ228037**, **FJ228038**, **FJ228081**

Luxembourg **DQ877749**, **EF563190**

Netherlands **AY423387**, **AY423383**, **AY877314**, **DQ877839**, **U34604**, **GQ399672**, **DQ877848**

Portugal **DQ663718**, **DQ663758**, **DQ666409**, **DQ666416**, **DQ877854**, **DQ877866**, **DQ877875**, **EF563195**

Slovenia **AJ971111**, **AJ971144**

Spain **AF256207**, **AJ006287**, **AY188561**, **AY315950**, **AY316009**, **AY541992**, **AY542131**, **AY833593**, **DQ103904**, **DQ878901**, **DQ878956**, **EF397445**, **EF583196**, **EF583217**, **EF583285**, **SP623200**, **EU255372**, **EU255417**, **EU255511**, **EU552228**, **EU786674**

Sweden **AF368317**, **AF378391**, **AF394468**, **AY165231**, **AY165277**, **DQ877891**

Switzerland **AF077679**, **AF316851**, **DQ877898**, **EF449825**, **EF449848**

United Kingdom **AF181126**, **AF494109**, **AY362127**, **DQ879060**, **EU236465**, **EU817055**
